# Associations Between Gender Gaps in Life Expectancy, Air Pollution, and Urbanization: A Global Assessment With Bayesian Spatiotemporal Modeling

**DOI:** 10.3389/ijph.2023.1605345

**Published:** 2023-05-10

**Authors:** Zhoupeng Ren, Shaobin Wang, Xianglong Liu, Qian Yin, Junfu Fan

**Affiliations:** ^1^ Institute of Geographic Sciences and Natural Resources Research, Chinese Academy of Sciences, Beijing, China; ^2^ State Key Laboratory of Resources and Environmental Information System, Institute of Geographic Sciences and Natural Resources Research, Chinese Academy of Sciences, Beijing, China; ^3^ School of Civil and Architectural Engineering, Shandong University of Technology, Zibo, Shandong, China

**Keywords:** air pollution, spatial heterogeneity analysis, urbanization, gender gaps in life expectancy, Bayesian spatiotemporal modeling

## Abstract

**Objectives:** It’s evident that women have a longer life expectancy than men. This study investigates the spatiotemporal trends of gender gaps in life expectancy (GGLE). It demonstrates the spatiotemporal difference of the influence factors of population-weighted air pollution (pwPM_2.5_) and urbanization on GGLE.

**Methods:** Panel data on GGLE and influencing factors from 134 countries from 1960 to 2018 are collected. The Bayesian spatiotemporal model is performed.

**Results:** The results show an obvious spatial heterogeneity worldwide with a continuously increasing trend of GGLE. Bayesian spatiotemporal regression reveals a significant positive relationship between pwPM_2.5_, urbanization, and GGLE with the spatial random effects. Further, the regression coefficients present obvious geographic disparities across space worldwide.

**Conclusion:** In sum, social-economic development and air quality improvement should be considered comprehensively in global policy to make a fair chance for both genders to maximize their health gains.

## Introduction

The gender differential in longevity has been a subject of interest for scientists. Generally, women have had longer life expectancy (LE) than men, though LE has steadily increased in both sexes during the last decades [1, 2]. Notably, the gap in gender-specific life expectancy (GGLE) is defined as the difference between LE of women and men, which has varied across space and time. For example, the GGLE were 6.9, 3.5, and 2.5 years in more developed, less developed, and least developed countries, respectively [3]. In addition, the female advantage in LE tends to be smaller among developing nations [4]. Meanwhile, the GGLE shows a difference across time. GGLE grew rapidly during most of the 20th century. Then, this trend was reversed, and the GGLE has declined since the 1980s. Prior papers found that physical health dimensions that cause morbidity and mortality are related to GGLE [5–8]. Furthermore, previous studies also indicated that income inequality and education level influence the GGLE [9, 10]. Accordingly, these facts mentioned above urge us not only to consider the gender gap in life expectancy but also to focus on the influencing factors of this spatial inequality.

Nevertheless, two knowledge gaps should be noted. First, the spatiotemporal trends of GGLE and the regional differences with the geospatial feature are largely unexplored at the global scale. Second, few studies have been performed to comprehensively investigate the environmental and social factors of GGLE and their spatiotemporal difference. For example, fine particulate matter (PM_2.5_), a major component of air pollution, is ranked in the top ten leading global risk factors for disease burdens [11]. Extensive studies have revealed that long-term exposure to PM_2.5_ is closely related to a reduction of LE at the country level [12–15] and cross-country level [16, 17]. Furthermore, urbanization plays a significant positive role in LE improvement [18]. A study in the U.S. also demonstrated that residents in metropolitan areas had larger gains in LE than those in non-metropolitan areas, which contributed to the widening gap [19]. Conversely, it has been found that urbanization may be an incubator for new epidemics and even pandemics that threaten human health worldwide [20, 21].

Accordingly, it is important to understand the spatiotemporal trends of GGLE and its environmental and social factors. Fortunately, Bayesian spatiotemporal modeling is a powerful tool for filling the gaps mentioned above by providing spatiotemporal patterns and producing smoothed maps to identify spatiotemporal trends and factors of public health levels [22]. Therefore, our current work examines the spatiotemporal patterns and variations of GGLE and its associations to PM_2.5_ pollution and urbanization from a global perspective with the potential effect of geographic space. Data on GGLE and influencing factors are collected from 134 countries from 1960 to 2018. The Bayesian spatiotemporal and Bayesian regression models are applied. Hence, our study could provide a new perspective on a spatiotemporal basis different from the traditional study. This paper could thus be expected to offer regional-specific implications for public health policymaking.

## Methods

### Variables

Life expectancy refers to the mean years of life remaining at a given age through the mortality rates observed at a given year [23]. This study considers the gap in gender-specific life expectancy (GGLE) as the dependent variable. Specifically, the value of GGLE is calculated as each country’s female life expectancy minus its male life expectancy (Table 1).

**TABLE 1:** Basic descriptions of the used variables (Worldwide, 1960 – 2018).

No.	Explanatory variables	Abbreviations	Unit
*Y*	Gender gaps in life expectancy at birth	GGLE	year
*Y*	Life expectancy at birth of male	LEm	year
*Y*	Life expectancy at birth of female	LEf	year
*X*_*1*_	Population-weighted PM_2.5_	pwPM_2.5_	μg/m^3^
*X*_*2*_	Urbanization population rate	urbanpop	% of total population
*X*_*3*_	Poverty gap index	poverty	-
*X*_*4*_	Primary education (% female)	education	% female
*X*_*5*_	Supply of calories	calories supply	kcal/capita/day
*X*_*6*_	Death rate from smoking	smoking	Annual number of deaths attributed to smoking per 100,000 people

We calculate the population-weighted PM_2.5_ (Table 1) at the country level as a proxy variable to quantify the magnitude of air pollution in the exposed population on the country scale by using the following formula:

$${pwPM}_{2.5}=\sum_{i=1}^{n}\left({PM}_{2.5}^{i}\times \frac{{pop}_{i}}{pop}\right)$$ (1)

In Eq. 1, ${pwPM}_{2.5}$ represents the population-weighted PM_2.5_ for a country; ${PM}_{2.5}^{i}$ refers to the value of ground-level PM_2.5_ in the ith grid. ${pop}_{i}$ is the population count in the ith grid; pop is the total population at country level. The annual concentrations of ground-level PM_2.5_ in the dataset are based on simulated aerosol optical depth [24].

The urbanization rate was found to be closely positively related to LE based on previous studies [25, 26]. Therefore, the urbanization rate is selected as one of the core independent variables in this study.

Several covariates were selected in our model, including poverty level, education level, nutrition level, and the death rate from smoking, which showed significant relations to LE based on existing studies. Poverty plays an important role in LE determinants [27, 28]. In this study, the poverty gap index was collected to reflect the level of poverty, which refers to the mean shortfall of consumption/income from the poverty line. Education level, especially for female education conditions, is an important factor in the LE [29, 30]. In this paper, we collected primary education level as the indicator to measure the education level, which refers to the female pupils as a percentage of total pupils at the primary level, including enrollments in public and private schools. Daily caloric supply refers to the average *per capita* caloric availability. This indicator indicates the caloric availability delivered to households but does not necessarily indicate the number of calories consumed. A prior study showed that food calories could increase LE [31]. The death rate from smoking (both sex and age-standardized) refers to the annual deaths attributed to smoking (per 100,000 people). Previous papers indicated the negative effects of smoking on LE [32–34].

### Data Sources

In this study, the data on life expectancy for females and males in each country from 1960 to 2018 are obtained from the World Development Indicators (WDIs) database published by the World Bank [35]. Further, the annual concentrations of ground-level PM_2.5_ (with dust and sea salt removed) data for 1998–2016 are collected from the Global Annual PM_2.5_ Grids data with the spatial resolution of 0.01°×0.01° released by EARTHDATA (https://sedac.ciesin.columbia.edu/data/set/sdei-global-annual-gwr-pm2-5-modis-misr-seawifs-aod). We obtained the gridded population of the world at 1,000 m spatial resolution for the year 2005, according to the Socioeconomic Data and Applications Center of NASA (https://sedac.ciesin.columbia.edu/data/collection/gpw-v4). Data on the share of urban population (% of the total population) is from the World Urbanization Prospects: 2018 Revision released by the United Nations Population Division. The data of the poverty gap index is from the Poverty and Inequality Platform by the World Bank, which is based on primary household survey data (https://data.worldbank.org/indicator). Data on the death rate from smoking (both sex with age-standardized) is from the Global Burden of Disease Collaborative Network by the Institute for Health Metrics and Evaluation (IHME) (http://ghdx.healthdata.org/gbd-results-tool). Data on daily caloric supply is from the Our World in Data (OWID) based on UN FAO & historical sources (https://ourworldindata.org/calorie-supply-sources). Data on education are collected from the database of the UNESCO Institute for Statistics (http://uis.unesco.org/).

### Statistical Analysis

To model GGLE, a series of Bayesian spatiotemporal models are proposed (Supplementary Table S1), where for each country (*i* = 1, 2, ..., 134) and year step (*t* = 1960,1961, ..., 2018), the GGLE, *y*_it_, was modeled by $y_{it}∼Normal\;\left(\mu_{it},\sigma_{e}^{2}\right)$ . Here, $\mu_{it}$ denotes the expected value of $y_{it}$ and $\sigma_{e}^{2}$ measures the variance of $y_{it}$ . The $\mu_{it}$ could be calculated as the following formula (model M0s):

$$\mu_{it}=\alpha +s_{i}+\theta_{t}+\delta_{it}$$ (2)

In Eq. 2, α is an intercept that measures the overall $y_{it}$ during the study period (1960–2018); The spatial term $s_{i}$ , denotes the spatial random effect capturing the spatial dependency of $y_{it}$ . We note that $s_{i}$ captures the overall spatial random effects common from 1960 to 2018, describes the difference between $y_{it}$ in the *i*-th country or region relative to the global average level, α . When $s_{i}$ >0, it indicates that the $y_{it}$ in country i is higher than overall $y_{it}$ across the whole study period. The term $\theta_{t}$ denotes a dynamic temporal trend that captures the overall temporal trend common to all countries. The $\delta_{it}$ is a space-time interaction random effect, representing a vector that varies through space and time [36]. The term $\delta_{it}$ allows spatial pattern changes from one time frame to another and temporal trend varies from one country to another.

Furthermore, a Bayesian spatiotemporal ecological regression was developed to investigate the impacts of air pollution and urbanization on GGLE and gender-specific life expectancy while adjusting for confounding factors and spatiotemporal variation. As an extension of model M0s, the variables of PM and UP (two interested variables) were considered, adjusting for confounding factors like poverty (P), education (E), calorie supply (C), and smoking (S). The new model was defined as the following formula (model M1s):

$$y_{it}=\alpha +s_{i}+\theta_{t}+\delta_{it}+\beta_{1}{\ln PM}_{it}+\beta_{2}{\ln UP}_{it}+\beta_{3}{\ln P}_{it}+\beta_{4}{\ln E}_{it}+\beta_{5}{\ln C}_{it}+\beta_{6}{\ln S}_{it}$$ (3)

In Eq. 3, $\beta_{1}$ , $\beta_{2}$ , ..., $\beta_{6}$ refer to the corresponding regression coefficients of pwPM_2.5_, urbanization, poverty, education, supply of calories, and smoking, respectively, measuring the effects of these six variables across 134 overall countries on y (GGLE, LEm, and LEf). A previous study found that the effects of air pollution and urbanization on LE and gender-specific LE may be spatially varied [37]. Model M1s is modified to allow each region to present its own regression coefficient to examine the spatially varied impacts of pwPM_2.5_ and urbanization on GGLE and gender-specific LE. In this paper, a total of 134 countries are divided into six regions based on the definition of the World Health Organization (WHO) (i.e., Africa, Americas, Eastern Mediterranean, Europe, South-East Asia, and Western Pacific), to investigate the region-specific effects of pwPM_2.5_ and urbanization on GGLE and gender-specific life expectancy, the modified regression model is shown as follow (model M2s):

$$y_{it}=\alpha +s_{i}\;\theta_{t}+\delta_{it}+\sum_{j=1}^{n}\beta_{1j}{\ln PM}_{it}+\sum_{j=1}^{n}\beta_{2j}{lnUP}_{it}+\beta_{3}{\ln P}_{it}+\beta_{4}{\ln E}_{it}+\beta_{5}{\ln C}_{it}+\beta_{6}{\ln S}_{it}$$ (4)

In Eq. 4, $\beta_{1j}$ and $\beta_{2j}$ represent the regression coefficients of influencing factors in a different region, j = 1, 2, …, n (n = 6). For detailed methods, please refer to the supplementary materials.

## Results

### Distribution and Variation Trends

**FIGURE 1:**
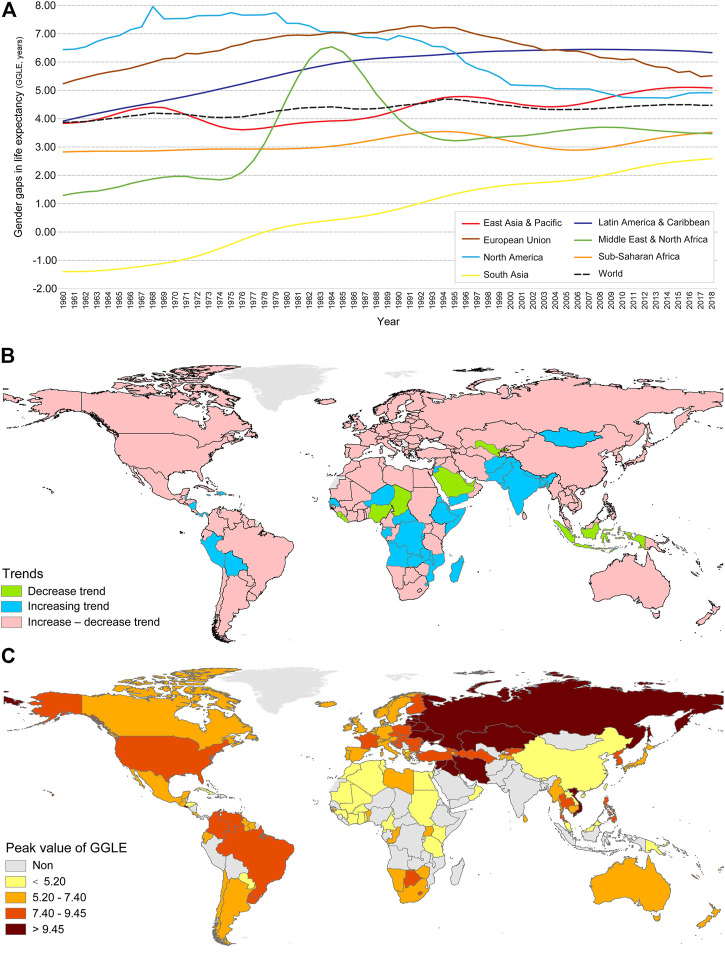
Variation, trends, and peak values of GGLE (Worldwide, 1960 – 2018). **(A)** Average GGLE in each region; **(B)** variation trends; **(C)** peak values of GGLE.

The average GGLE at the country level and the world is illustrated from 1960 to 2018 (Figure 1). Some key points can be drawn. Firstly, we could find the spatial difference across regions. Europe and North American countries show the highest peak values of GGLE, which shows a trend of rising first and then falling in GGLE. Secondly, other regions show an increasing trend during the study period, similar to the global trend. Furthermore, GGLE in South Asia (e.g., India) shows an increasing trend from negative to positive values, whereas other countries show positive values of GGLE during the study period. In addition, the GGLE in the Middle East and North Africa presents an anomaly peak during the 1980s, possibly related to the sharp decreased male LE values in Iran and Iraq.

Further, most countries show a trend from increase to decrease of GGLE with peak values, 41 countries have an increasing trend without a peak value, and only 12 countries/regions show a decreasing trend without a peak value during the study period. The difference is depicted in space, indicating that the countries with increasing or decreasing trends are mainly concentrated in South Asia, Middle Africa, the Middle East, and Latin America (Figure 1). Countries with peak values also present spatial differences. For example, Russia and countries in East Europe, Central Asia, Middle East show the higher level of peak values of GGLE, and countries in Western Europe, America, South Africa, Japan, South Korea, Australia, and New Zealand show the middle range of peak values of GGLE, while countries in North Africa, East-southeastern Asia show the lower peak values of GGLE (Figure 1).

### Spatial Pattern and Its Changes Over Time

**FIGURE 2:**
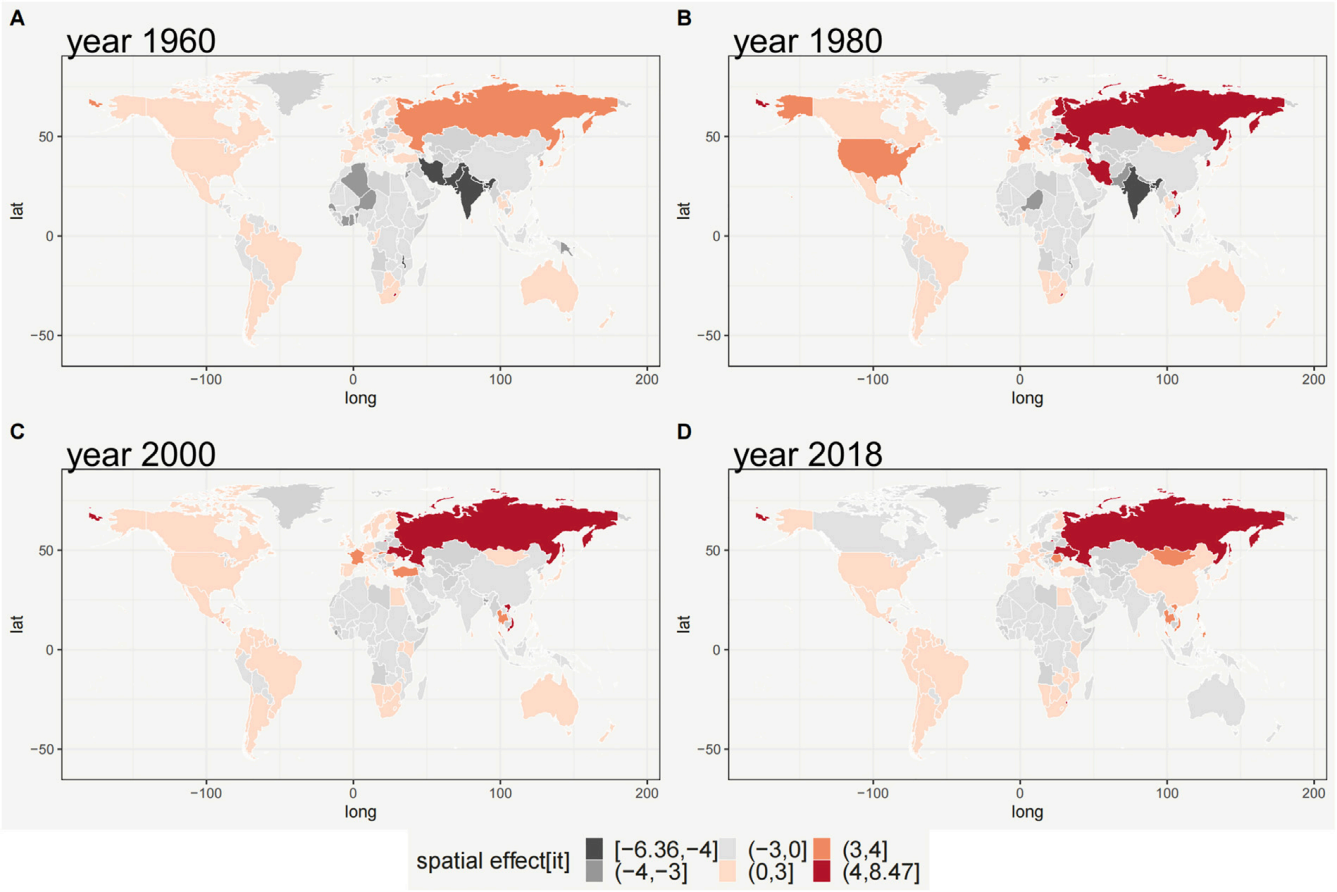
The combined spatial effects (s_i_+θ_t_+δ_it_) of GGLE (Worldwide, 1960, 1980, 2000, 2018). **(A)** 1960; **(B)** 1980; **(C)** 2000; **(D)** 2018.

The combined spatial effects of $s_{i}+\theta_{t}+\delta_{it}$ at four different time points (1960, 1980, 2000, and 2018) for GGLE were estimated using the Bayesian spatiotemporal modeling, which depicts a spatial pattern and its changes during the study period 1960–2018 (Figure 2). Positive values of the combined spatial effects indicate that GGLE in country *i* at time point *t* is higher than the overall level, while negative values indicate that lower than the overall level. We found that the spatial pattern of GGLE slightly changed from 1960 to 2018. However, the whole spatial pattern is similar at these four-time points. For example, GGLE in Russia is consistently higher than the overall level. In contrast, most countries in Africa have lower GGLE than the overall level. China has a substantial change in GGLE from 1960 to 2018, which indicates a lower level in 1960, 1980, and 2000, but higher levels in 2018 compared to the overall level in the world. Other countries like the U.S., Japan, South Korea, Brazil, Argentina, and South Africa show that GGLE is consistently higher than the overall level.

### Overall and Local Temporal Trends

**FIGURE 3:**
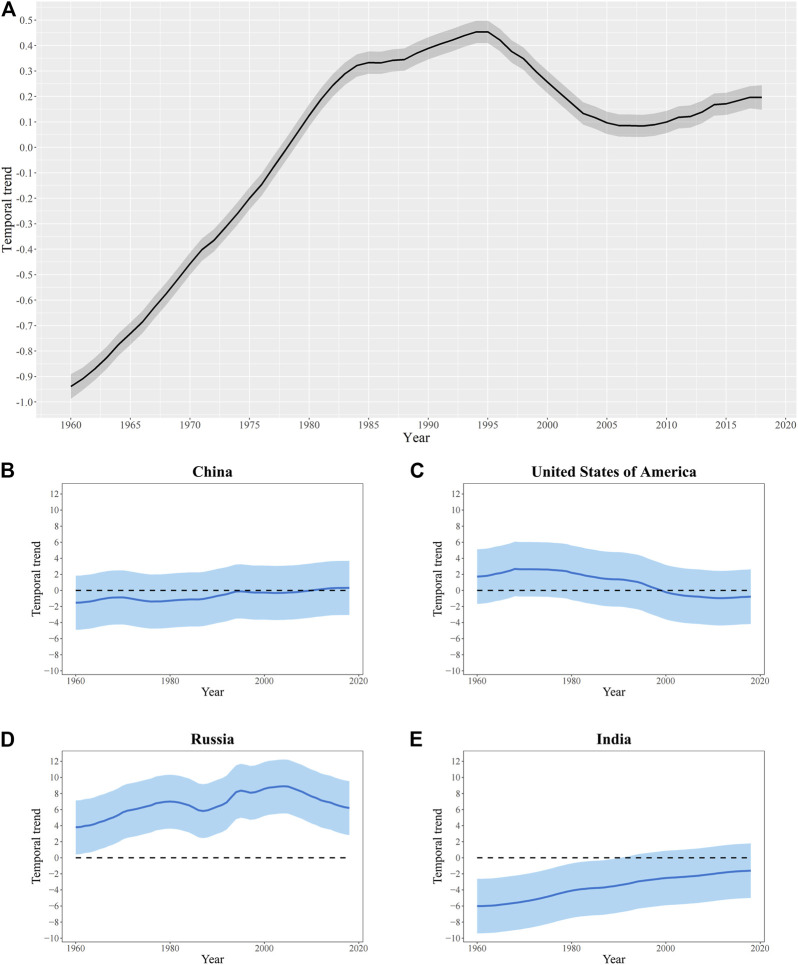
The temporal trend of GGLE which shows a probability of 95% (Worldwide, China, the U.S., Russia, and India, 1960 – 2018). **(A)** Worldwide; **(B)** China; **(C)** the U.S.; **(D)** Russia; **(E)** India.

The overall temporal trend of GGLE ( $\theta_{t}$ ) is estimated using the Bayesian spatiotemporal model from 1960 to 2018. The overall temporal trend of GGLE presents a clear nonlinear temporal trend in the world (Figure 3). The GGLE increased rapidly from 1960 to 1985, and then grew slowly down but maintained an increasing trend. From 1995 to 2005, the GGLE decreased rapidly by a clear linear temporal trend. After 2005, the GGLE increased again. Moreover, we estimated the country-specific temporal trends ( $\theta_{t}+\delta_{it}$ ) of GGLE to explore whether there are huge differences across countries worldwide. Figure 3 shows temporal trends of GGLE in four selected countries from 1960 to 2018, including China, the U.S., Russia, and India. China slightly increases GGLE, while GGLE in the U.S. shows a nonlinear temporal trend. However, there are no clear significant temporal trends of GGLE in China and the U.S. during the study period. Figure 3 indicates that GGLE in Russia has a stronger upward trend from 1960 to 2005 but a clear downward trend from 2005 to 2018. India shows a significant increase in temporal trend. Our findings demonstrate apparent spatial differences in the local temporal trends across countries worldwide.

### Influence of Air Pollution and Urbanization Factors

The global (Table 2) effects of air pollution and urbanization are investigated based on Bayesian regression modeling. The estimate of parameter φ suggested that the spatially structured component explained 7.7% of the variance in the overall spatial effects of GGLE (Table 2). However, the smaller DIC and WAIC scores derived from models with spatial random effects (Table 2) indicated that the spatial structure considered in the Bayesian spatiotemporal regression models could improve the data fitness. Several key findings could be observed. First, a significant positive relationship can be seen between pwPM_2.5_ and GGLE, and no significant relations are found between pwPM_2.5_ and LE in males, and females can be found. Therefore, considering spatial random effects on the global model, the GGLE increased by 0.00034% when a one percent increase in pwPM_2.5_. Second, urbanization shows a significant positive relation to GGLE with spatial random effects, and significant positive relations are found between urbanization and LE in males and females as well (Table 2). Third, significant negative relations between poverty and GGLE can be found, while significant positive relations between calories supply, smoking, and GGLE can be identified.

**TABLE 2:** Global regression parameters of GGLE/male/female life expectancy, air pollution, urbanization, and control variables, estimated by posterior means of the Bayesian regression model with 95% credible intervals (Worldwide, 1960 – 2018).

	GGLE	LEm	LEf
Fixed effects			
(Intercept)	−3.026 (−4.119, −1.932)	1.979 (1.714, 2.244)	1.839 (1.574, 2.104)
pwPM2.5	0.034* (0.019, 0.048)	−0.003 (−0.007, 0.001)	−0.001 (−0.004, 0.003)
Urbanpop	0.251* (0.174, 0.328)	0.147* (0.128, 0.165)	0.153* (0.134, 0.172)
Poverty	−0.012* (−0.019, −0.005)	0.006* (0.004, 0.007)	0.005* (0.003, 0.007)
Education	−0.061 (−0.267, 0.146)	0.324* (0.274, 0.374)	0.308* (0.258, 0.358)
Calories supply	0.419* (0.304, 0.533)	0.083* (0.055, 0.111)	0.109* (0.082, 0.137)
Smoking	0.076* (0.024, 0.127)	−0.065* (−0.078, −0.053)	−0.057* (−0.070, −0.044)
Random effects (hyperparameters)			
$\tau_{e}$ (Precision of Gaussian error)	156 (127.62, 191)	2,530 (1910.37, 3,210)	2,860 (2,280, 3,550)
$\tau_{s}$ (marginal precision of BYM2)	5.11 (4.22, 6.14)	76.0 (58.4, 98.0)	79.4 (56.7, 109.0)
φ (mixing parameter of BYM2)	0.473 (0.372, 0.572)	0.665 (0.540, 0.769)	0.648 (0.456, 0.801)
$\tau_{\theta }$ (precision of $\theta_{t}$ )	20,400 (7,163.735, 47,400)	60,400 (29,610.45, 110,000)	65,400 (30,500, 121,000)
$\tau_{\delta }$ (precision of space-time interaction)	180 (142.163, 222)	3,330 (2,398.57, 4,670)	28,500 (22,800, 35,400)
Goodness of fit			
DIC	−4,433.95	−11,608.70	−11,726.27
WAIC	−4,307.81	−11,521.02	−11,698.52

Further, the six WHO regions exhibit geographic disparities in the effects of pwPM_2.5_ and urbanization on GGLE and gender-specific life expectancy (Table 3). First, our results indicate that the negative effect of pwPM_2.5_ on GGLE can be found in Africa, Eastern Mediterranean, and South-East Asia. A positive effect of pwPM_2.5_ on GGLE is significant in America, Europe, and Western Pacific. No significant relations between pwPM_2.5_ and LE in males and females are found in the six regions. Second, our results indicate that the negative effect of urbanization on GGLE can be found in Africa and Europe. In contrast, the positive effect of urbanization on GGLE is significant in America and Eastern Mediterranean. Significant positive relations between urbanization and LE in males and females are found in the six regions.

**TABLE 3:** Local regression parameters of GGLE/male/female life expectancy, air pollution, and urbanization estimated by posterior means of the Bayesian regression model with spatial random effects with 95% credible intervals (Six WHO regions, 1960 – 2018).

Region	pwPM_2.5_			Urbanization		
	GGLE	LEm	LEf	GGLE	LEm	LEf
Africa	−0.036* (−0.066, −0.007)	0.003 (−0.006, 0.012)	0.005 (−0.008, 0.018)	−0.047* (−0.079, −0.017)	0.245* (0.213, 0.275)	0.164* (0.132, 0.197)
America	0.041* (0.014, 0.067)	−0.002 (−0.006, 0.002)	−0.001 (−0.005, 0.004)	0.054* (0.026, 0.082)	0.131* (0.103, 0.158)	0.156* (0.137, 0.176)
Eastern Mediterranean	−0.297* (−0.382, −0.214)	−0.002 (−0.013, 0.007)	−0.007 (−0.024, 0.011)	0.070* (0.025, 0.120)	0.098* (0.053, 0.140)	0.139* (0.113, 0.165)
Europe	0.198* (0.152, 0.244)	0.003 (−0.005, 0.013)	0.015 (0.002, 0.029)	−0.054* (−0.089, −0.021)	0.091* (0.049, 0.132)	0.138* (0.114, 0.162)
South-East Asia	−0.114* (−0.162, −0.065)	0.001 (−0.008, 0.011)	0.003 (−0.014, 0.020)	−0.011 (−0.058, 0.035)	0.049* (0.012, 0.086)	0.125* (0.091, 0.157)
Western Pacific	0.077* (0.059, 0.096)	−0.001 (−0.007, 0.004)	−0.004 (−0.009, 0.002)	−0.019 (−0.050, 0.011)	0.066* (0.032, 0.100)	0.140* (0.115, 0.164)

## Discussion

It has been largely unexplored the spatial distribution and variation of GGLE worldwide. The current study is one of the first that exhibited the spatiotemporal trends in GGLE and examined the effects of air pollution and urbanization on GGLE and gender-specific life expectancy from a global perspective. Further, the geographic disparities in the impacts of these influencing factors on GGLE among six WHO regions can be quantified by the regression coefficients by Bayesian spatiotemporal ecological regression. Several key points can be further discussed as follows.

First, the spatiotemporal trends in GGLE at the global scale are investigated in this study. Some previous studies have been performed on GGLE’s distribution at the cross-country level. It was found that GGLE grew rapidly during most of the 20th century. Then, a reversal of this trend has been observed that the GGLE has declined since the 1980s among many developed countries such as Italy [38], France [39], Netherlands [40], Sweden and Japan [9, 41], Canada [42], South Korea [43], and the United States [44]. It also revealed that GGLE trend showed inverted U-curves in most Organization for Economic Co-operation and Development (OECD) countries undergoing three phases, i.e., growth, peak and stability, and decline [45]. Further, it was found that GGLE varied substantially in EU 28 Member States in 2015, which showed that GGLE was 5.4 years on average [46]. In addition, it was found that the GGLE converges in highly developed countries but diverges in less developed countries [47]. Our findings extend the study on the spatial distribution of GGLE worldwide, forming an evident spatial heterogeneity with a continuously increasing trend of GGLE from 1960 to 2018. Different from previous studies, our results decomposed the spatiotemporal variability of GGLE into three components: the common spatial pattern across the study periods, the overall temporal trend, and local temporal trends. For one thing, the average values of GGLE among regions in Africa and South-East Asia show much lower GGLE, and the Americas, Europe, and Western Pacific exhibit a relatively higher level compared to the global level. For another, most countries present the increase–decrease trend with the peak value of GGLE, similar to the patterns of OECD countries. Further, the distribution of the peak values of GGLE shows similar patterns to the average values of GGLE in the world. In comparison, only 27% of countries show continuous increasing or decreasing trends without a peak GGLE value. These countries are the least developed countries located in Africa, Asia, and Latin America. In sum, our findings indicate that the average level and peak values of GGLE show obvious differences among six WHO regions in the world.

Second, it is evident that women have longer LE than men, and female superiority in LE appears to be one of the most striking features of human health [48–50]. Several factors, such as biological [51] and behavioral factors [52], have been proposed to explain these differences. However, these factors cannot fully explain why GGLE would fluctuate over time [50]. Importantly, social-economic and environmental factors have not been taken into consideration in the gender differences, though these factors are closely related to LE, which have been investigated in extensive studies [12, 53–56]. A previous study probed the difference of social-economic determinants in LE between total LE and female LE, which showed that the fit of the models for the female population is weaker than the respective fits of the models for the entire population, indicating that the parameters should be different in female LE [57]. A recent study revealed the spatial heterogeneity of the relations between LE and *per capita* GDP and pwPM_2.5_ based on the Bayesian regression model [58]. This paper introduces pwPM_2.5_ and urbanization factors as our core independent variables. Using the Bayesian spatiotemporal ecological regression, our findings reveal the significant negative influence of pwPM2.5 and urbanization on GGLE with spatial random effects. Although no significant relations are found between pwPM_2.5_ and LE in males and females, we reveal the negative regression coefficients in females are lower than in males. In general, our finding is in line with previous studies, which showed that adult males had higher exposure to air pollutant factors than females [59–61]. Thus, the current paper indicates that the negative impacts of air pollution on males are more severe than on females, which may positively relate to GGLE. Meanwhile, we find a significant positive influence of urbanization on GGLE with spatial random effects. Urban populations have longer life expectancies than rural populations. A previous study in the U.S. showed that urban life expectancy increased in women faster than men, whereas rural life expectancy decreased in men faster than women [62]. Our findings are consistent with the previous paper that the urbanization factor may be more advantageous to females than males, which may further result in the significant positive influence of urbanization on GGLE.

Moreover, our findings could provide several implications. Firstly, the average level and peak values of GGLE show obvious differences among WHO regions in the world, and the countries with higher levels of GGLE are mostly related to higher development levels. It could be inferred that the GGLE may keep increasing with social-economic development and worsening air pollution in most regions worldwide. Secondly, it further indicates that the air pollution factor with spatial autocorrelation should consider the influence of GGLE. Further investigations are needed to probe the gender differences in PM_2.5_ pollution. Our modeling presents obvious geographic disparities of the impacts of PM_2.5_ across space. America, Europe, and Western Pacific showed positive relations between pwPM_2.5_ and GGLE, which is in line with the global trend. Interestingly, these regions exhibit a relatively higher level than the global average. In contrast, Africa, Eastern Mediterranean, and South-East Asia showed the opposite trend, indicating that spatial heterogeneity should be highlighted, especially in the world’s developing regions. Similarly, the spatial heterogeneity of urbanization influence should also be focused on the regions such as Africa, America, Eastern Mediterranean, and Europe. Furthermore, the coupling relationship between urbanization and air pollution has become a hotspot internationally in sustainable development studies [63]. Developing sustainable urbanization practices with air pollution control is crucial for addressing sustainable development [64]. Our findings further indicated that air pollution and urbanization factors should be considered comprehensively in achieving Sustainable Development Goals and the influencing mechanism of GGLE. Therefore, our investigation may provide a potential solution to narrow the GGLE by coordinating the roles of social-economic growth and improvement of air quality in a society with a fair chance for both men and women to maximize their gaps in life expectancy.

Nevertheless, several limitations and study prospects in this paper should be noted. Firstly, although more accurate human PM_2.5_ exposure data are much needed in the future, the error associated with satellite and simulated PM_2.5_ concentration was assessed and calibrated in this paper. Similarly, we used the death rate from smoking rather than smoking prevalence as a covariate due to the limitation of data sources, which could be improved in future studies. Secondly, the effect of PM_2.5_ and urbanization may be biased due to omitted confounders and spatial confounding [65]. The spatial confounding indicates that unobserved influencing factors may confound the model estimates if they directly impact GGLE and, at the same time, are correlated with the observed covariates. Therefore, this issue should be fully considered to improve the accuracy of model estimation and spatial modeling [66]. Thirdly, the influence of PM_2.5_ and urbanization indicated that the confounders for GGLE are not the same as for life expectancy. Hence, it may need further analysis of GGLE rather than a comparison of the coefficients between males and females. Fourthly, the collinearity results due to the multi-environmental and social-economic factors should be interpreted with caution in ecological studies [67], especially when more influencing factors and covariates are selected in future papers. Further, unaccounted confounders mentioned above make policy inferences quite difficult. Accordingly, follow-up studies can be performed to fully recognize the mechanism of the spatiotemporal trends in GGLE, especially on the global scale, though several factors have been probed, such as gender inequality [46], Human Development Index [45], invest in health and the gender gap in wages [44], women’s status and modernization [10], diseases [68], alcohol and life satisfaction [69], homicides [70], etc. Fifthly, although our study reveals the nonlinearity in the trend in GGLE, the driving mechanism still needs to be thoroughly probed based on the nonlinear hypothesis. Last, this paper only used historical data in our modeling. Hence, prediction and simulation of GGLE variation in space could provide in-depth information and policy implication to meet the future needs of aging societies and improve sustainability in the coming years.

## Abbreviations

BYM, Besag-York-Mollié model; DIC, Deviance information criterion; GGLE, Gender gaps in life expectancy; INLA, Integrated nested Laplace approximation; LE, Life expectancy; OECD, Organization for Economic Co-operation and Development; pwPM_2.5_, Population-weighted concentration of fine inhalable particles; VIF, Variance inflation factors; WAIC, Watanabe-Akaike information criterion; WHO, World Health Organization.

## References

[1] Oeppen J Vaupel JW. Demography. Broken Limits to Life Expectancy. Science (2002) 296:1029–31. 10.1126/science.1069675 12004104

[2] Vaupel JW. Biodemography of Human Ageing. Nature (2010) 464:536–42. 10.1038/nature08984 20336136 PMC4010874

[3] United Nations. Department of Economic and Social Affairs, Population Division. World Mortality Report 2009. New York: United Nations publications (2012).

[4] Nathanson CA. Sex Differences in Mortality. Annu Rev Sociol (1984) 10:191–213. 10.1146/annurev.so.10.080184.001203 12339750

[5] Lai DJ Tarwater PM Hardy RJ. Measuring the Impact of HIV/AIDS, Heart Disease and Malignant Neoplasms on Life Expectancy in the USA from 1987 to 2000. Public Health (2006) 120:486–92. 10.1016/j.puhe.2005.12.009 16730037

[6] Pinkhasov RM Shteynshlyuger A Hakimian P Lindsay GK Samadi DB Shabsigh R. Are Men Shortchanged on Health? Perspective on Life Expectancy, Morbidity, and Mortality in Men and Women in the United States. Int J Clin Pract (2010) 64:465–74. 10.1111/j.1742-1241.2009.02289.x 20456193

[7] Chen H Zhou Y Sun L Chen Y Qu X Chen H Non-communicable Diseases Are Key to Further Narrow Gender gap in Life Expectancy in Shanghai, China. BMC Public Health (2020) 20:839. 10.1186/s12889-020-08932-x 32493253 PMC7268263

[8] Kiadaliri A. Avoidable Deaths in Sweden, 1997–2018: Temporal Trend and the Contribution to the Gender gap in Life Expectancy. BMC Public Health (2021) 21:519. 10.1186/s12889-021-10567-5 33731076 PMC7968161

[9] Trovato F Heyen NB. A Divergent Pattern of the Sex Difference in Life Expectancy: Sweden and Japan, Early 1970s‐late 1990s. Soc Biol (2003) 50:238–58. 10.1080/19485565.2003.9989074 16382814

[10] Clark R Peck BM. Examining the Gender Gap in Life Expectancy: A Cross-National Analysis, 1980–2005. Soc Sci Q (2012) 93:820–37. 10.1111/j.1540-6237.2012.00881.x

[11] Roth GA Abate D Abate KH Abay SM Abbafati C Abbasi N Global, Regional, and National Age-sex-specific Mortality for 282 Causes of Death in 195 Countries and Territories, 1980–2017: a Systematic Analysis for the Global Burden of Disease Study 2017. Lancet (2018) 392:1736–88. 10.1016/S0140-6736(18)32203-7 30496103 PMC6227606

[12] Pope CA Ezzati M Dockery DW. Fine-particulate Air Pollution and Life Expectancy in the United States. N Engl J Med (2009) 360:376–86. 10.1056/NEJMsa0805646 19164188 PMC3382057

[13] Correia AW Pope CA Dockery DW Wang Y Ezzati M Dominici F. Effect of Air Pollution Control on Life Expectancy in the United States: an Analysis of 545 U.S. Counties for the Period from 2000 to 2007. Epidemiology (2013) 24:23–31. 10.1097/EDE.0b013e3182770237 23211349 PMC3521092

[14] Etchie TO Etchie AT Adewuyi GO Pillarisetti A Sivanesan S Krishnamurthi K The Gains in Life Expectancy by Ambient PM2.5 Pollution Reductions in Localities in Nigeria. Environ Pollut (2018) 236:146–57. 10.1016/j.envpol.2018.01.034 29414335

[15] Wu Y Wang W Liu C Chen R Kan H. The Association between Long-Term fine Particulate Air Pollution and Life Expectancy in China, 2013 to 2017. Sci Total Environ (2020) 712:136507. 10.1016/j.scitotenv.2020.136507 32050378

[16] Sarkodie SA Strezov V Jiang Y Evans T. Proximate Determinants of Particulate Matter (PM2.5) Emission, Mortality and Life Expectancy in Europe, Central Asia, Australia, Canada and the US. Sci Total Environ (2019) 683:489–97. 10.1016/j.scitotenv.2019.05.278 31141750

[17] Bu X Xie Z Liu J Wei L Wang X Chen M Global PM2.5-attributable Health burden from 1990 to 2017: Estimates from the Global Burden of Disease Study 2017. Environ Res (2021) 197:111123. 10.1016/j.envres.2021.111123 33823194

[18] Kim JI Kim G Kwak S Baek K Na G Kim JH The Settings, Pros and Cons of the New Surgical Robot da Vinci Xi System for Transoral Robotic Surgery (TORS): A Comparison With the Popular da Vinci Si System. Soc Indic Res (2016) 129:391–6. 10.1097/SLE.0000000000000313 27661201

[19] Singh GK Siahpush M. Widening Rural–Urban Disparities in Life Expectancy, U.S., 1969–2009. Am J Prev Med (2014) 46:e19–e29. 10.1016/j.amepre.2013.10.017 24439358

[20] Patel RB Burke TF. Urbanization — an Emerging Humanitarian Disaster. New Engl J Med (2009) 361:741–3. 10.1056/NEJMp0810878 19692687

[21] Neiderud C-J. How Urbanization Affects the Epidemiology of Emerging Infectious Diseases. Infect Ecol Epidemiol (2015) 5:27060. 10.3402/iee.v5.27060 26112265 PMC4481042

[22] Li J Liang J Wang J Ren Z Yang D Wang Y Spatiotemporal Trends and Ecological Determinants in Maternal Mortality Ratios in 2,205 Chinese Counties, 2010–2013: A Bayesian Modelling Analysis. PLOS Med (2020) 17:e1003114. 10.1371/journal.pmed.1003114 32413025 PMC7228041

[23] Shryock HS Siegel JS Stockwell EG. The Methods and Materials of Demography. Condensed ed. New York: Academic Press (1976).

[24] Hammer MS van Donkelaar A Li C Lyapustin A Sayer AM Hsu NC Global Estimates and Long-Term Trends of fine Particulate Matter Concentrations (1998–2018). Environ Sci Technol (2020) 54:7879–90. 10.1021/acs.est.0c01764 32491847

[25] Bergh A Nilsson T. Good for Living? on the Relationship between Globalization and Life Expectancy. World Dev (2010) 38:1191–203. 10.1016/j.worlddev.2010.02.020

[26] Shahbaz M Loganathan N Mujahid N Ali A Nawaz A. Determinants of Life Expectancy and its Prospects under the Role of Economic Misery: A Case of Pakistan. Soc Indic Res (2016) 126:1299–316. 10.1007/s11205-015-0927-4

[27] Regidor E Calle ME Navarro P Domínguez V. Trends in the Association between Average Income, Poverty and Income Inequality and Life Expectancy in Spain. Soc Sci Med (2003) 56:961–71. 10.1016/S0277-9536(02)00107-7 12593870

[28] Riumallo-Herl C Canning D Salomon JA. Measuring Health and Economic Wellbeing in the Sustainable Development Goals Era: Development of a Poverty-free Life Expectancy Metric and Estimates for 90 Countries. Lancet Glob Health (2018) 6:e843–58. 10.1016/S2214-109X(18)30277-8 30012266

[29] Crimmins EM Saito Y. Trends in Healthy Life Expectancy in the United States, 1970–1990: Gender, Racial, and Educational Differences. Soc Sci Med (2001) 52:1629–41. 10.1016/s0277-9536(00)00273-2 11327137

[30] Hoque MM King EM Montenegro CE Orazem PF. Revisiting the Relationship between Longevity and Lifetime Education: Global Evidence from 919 Surveys. J Popul Econ (2019) 32:551–89. 10.1007/s00148-018-0717-9

[31] Sufian AJM. Socio-Economic Correlates of Life Expectancy at Birth: The Case of Developing Countries. J Popul Health Stud (1989) 9:214–26. 12342756

[32] Rogot E Sorlie PD Johnson NJ. Life Expectancy by Employment Status, Income, and Education in the National Longitudinal Mortality Study. Public Health Rep (1992) 107:457–61. 1641443 PMC1403677

[33] Allen RT Hales NM Baccarelli A Jerrett M Ezzati M Dockery DW Countervailing Effects of Income, Air Pollution, Smoking, and Obesity on Aging and Life Expectancy: Population-Based Study of U.S. Counties. Environ Health (2016) 15:86. 10.1186/s12940-016-0168-2 27520789 PMC4983078

[34] Manuel DG Perez R Sanmartin C Taljaard M Hennessy D Wilson K Measuring burden of Unhealthy Behaviours Using a Multivariable Predictive Approach: Life Expectancy Lost in Canada Attributable to Smoking, Alcohol, Physical Inactivity, and Diet. PLoS Med (2016) 13:e1002082. 10.1371/journal.pmed.1002082 27529741 PMC4986987

[35] World Bank. World Development Indicators. Washington, DC: The World Bank Group (2019).

[36] Johnson DP Ravi N Braneon CV. Spatiotemporal Associations between Social Vulnerability, Environmental Measurements, and COVID-19 in the Conterminous United States. GeoHealth (2021) 5:e2021GH000423. 10.1029/2021GH000423 PMC8335698 34377879

[37] Wang S Ren Z Liu X Yin Q. Spatiotemporal Trends of Life Expectancy, Economic Growth, and Air Pollution: A 134 Countries Investigation Based on Bayesian Modeling. Soc Sci Med (2021) 114660. 10.1016/j.socscimed.2021.114660 34953418

[38] Conti S Farchi G Masocco M Minelli G Toccaceli V Vichi M. Gender Differentials in Life Expectancy in Italy. Eur J Epidemiol (2003) 18:107–12. 10.1023/A:1023029618044 12733831

[39] Meslé F. Écart d’espérance de vie entre les sexes: les raisons du recul de l’avantage féminin. Revue d’Épidémiol et de Santé Publique (2004) 52:333–52. 10.1016/S0398-7620(04)99063-3 15480291

[40] Spijker J van Poppel F van Wissen L. Explaining New Trends in the Gender gap of Mortality: Insights from a Regional Trend-Analysis of the Netherlands. Vienna Yearb Popul Res (2007) 5:61–92. 10.1553/populationyearbook2007s61

[41] Sundberg L Agahi N Fritzell J Fors S. Why Is the Gender gap in Life Expectancy Decreasing? the Impact of Age- and Cause-specific Mortality in Sweden 1997–2014. Int J Public Health (2018) 63:673–81. 10.1007/s00038-018-1097-3 29654335 PMC6015620

[42] Trovato F Lalu N. From Divergence to Convergence: The Sex Differential in Life Expectancy in Canada, 1971–2000. Can Rev Sociol Revue canadienne de Sociol (2007) 44:101–22. 10.1111/j.1755-618X.2007.tb01149.x 17644998

[43] Yang S Khang Y-H Chun H Harper S Lynch J. The Changing Gender Differences in Life Expectancy in Korea 1970–2005. Soc Sci Med (2012) 75:1280–7. 10.1016/j.socscimed.2012.04.026 22739261

[44] Leung MCM Zhang J Zhang J. An Economic Analysis of Life Expectancy by Gender with Application to the United States. J Health Econ (2004) 23:737–59. 10.1016/j.jhealeco.2003.11.001 15587696

[45] Liu Y Arai A Kanda K Lee RB Glasser J Tamashiro H. Gender Gaps in Life Expectancy: Generalized Trends and Negative Associations with Development Indices in OECD Countries. Eur J Public Health (2013) 23:563–8. 10.1093/eurpub/cks049 22542541

[46] Kolip P Lange C. Gender Inequality and the Gender gap in Life Expectancy in the European Union. Eur J Public Health (2018) 28:869–72. 10.1093/eurpub/cky076 29767703

[47] Medalia C Chang VW. Gender equality, Development, and Cross-National Sex Gaps in Life Expectancy. Int J Comp Sociol (2011) 52:371–89. 10.1177/0020715211426177

[48] Barford A Dorling D Davey Smith G Shaw M. Life Expectancy: Women Now on Top Everywhere. BMJ (2006) 332:808. 10.1136/bmj.332.7545.808 16601021 PMC1432200

[49] Luy M Minagawa Y. Gender Gaps--Life Expectancy and Proportion of Life in Poor Health. Health Rep (2014) 25:12–9. 25517936

[50] Hossin MZ. The Male Disadvantage in Life Expectancy: Can We Close the Gender gap? Int Health (2021) 13:482–4. 10.1093/inthealth/ihaa106 33533409 PMC7928849

[51] Nakamura E Miyao K. Sex Differences in Human Biological Aging. J Gerontol Ser A (2008) 63:936–44. 10.1093/gerona/63.9.936 18840798

[52] Wingard DL. The Sex Differential in Morbidity, Mortality, and Lifestyle. Annu Rev Public Health (1984) 5:433–58. 10.1146/annurev.pu.05.050184.002245 6372818

[53] Preston SH. The Changing Relation between Mortality and Level of Economic Development. Popul Stud (1975) 29:231–48. 10.1080/00324728.1975.10410201 11630494

[54] Wilkinson RG. Income Distribution and Life Expectancy. BMJ : Br Med J (1992) 304:165–8. 10.1136/bmj.304.6820.165 1637372 PMC1881178

[55] Wang S Luo K Liu Y Zhang S Lin X Ni R Economic Level and Human Longevity: Spatial and Temporal Variations and Correlation Analysis of Per Capita GDP and Longevity Indicators in China. Arch Gerontol Geriatr (2015) 61:93–102. 10.1016/j.archger.2015.03.004 25847813

[56] de Keijzer C Agis D Ambrós A Arévalo G Baldasano JM Bande S The Association of Air Pollution and Greenness with Mortality and Life Expectancy in Spain: A Small-Area Study. Environ Int (2017) 99:170–6. 10.1016/j.envint.2016.11.009 27871798

[57] Mazumdar K. Improvements in Life Expectancy: 1960–1995 an Exploratory Analysis. Soc Indic Res (2001) 55:303–28. 10.1023/A:1010934906630

[58] Wang S Ren Z Liu X Yin Q. Spatiotemporal Trends in Life Expectancy and Impacts of Economic Growth and Air Pollution in 134 Countries: A Bayesian Modeling Study. Soc Sci Med (2022) 293:114660. 10.1016/j.socscimed.2021.114660 34953418

[59] Chen D Mayvaneh F Baaghideh M Entezari A Ho HC Xiang Q Utilizing Daily Excessive Concentration Hours to Estimate Cardiovascular Mortality and Years of Life Lost Attributable to fine Particulate Matter in Tehran, Iran. Sci Total Environ (2020) 703:134909. 10.1016/j.scitotenv.2019.134909 31757557

[60] Hu Y Yao M Liu Y Zhao B. Personal Exposure to Ambient PM2.5, PM10, O3, NO2, and SO2 for Different Populations in 31 Chinese Provinces. Environ Int (2020) 144:106018. 10.1016/j.envint.2020.106018 32771828

[61] Zhang Y. All-cause Mortality Risk and Attributable Deaths Associated with Long-Term Exposure to Ambient PM2. 5 in Chinese Adults. Environ Sci Technol (2021) 55:6116–27. 10.1021/acs.est.0c08527 33870687

[62] Abrams LR Myrskylä M Mehta NK. The Growing Rural–Urban divide in US Life Expectancy: Contribution of Cardiovascular Disease and Other Major Causes of Death. Int J Epidemiol (2021) 50:1970–8. 10.1093/ije/dyab158 34999859 PMC8743112

[63] Fan W Wang H Liu Y Liu H. Spatio-temporal Variation of the Coupling Relationship between Urbanization and Air Quality: A Case Study of Shandong Province. J Clean Prod (2020) 272:122812. 10.1016/j.jclepro.2020.122812

[64] Ochoa JJ Tan Y Qian QK Shen L Moreno EL. Learning from Best Practices in Sustainable Urbanization. Habitat Int (2018) 78:83–95. 10.1016/j.habitatint.2018.05.013

[65] Hodges JS Reich BJ. Adding Spatially-Correlated Errors Can Mess up the Fixed Effect You Love. Am Stat (2010) 64:325–34. 10.1198/tast.2010.10052

[66] Mäkinen J Numminen E Niittynen P Luoto M Vanhatalo J. Spatial Confounding in Bayesian Species Distribution Modeling. Ecography (2022) 2022:e06183. 10.1111/ecog.06183

[67] Crouse DL Peters PA Hystad P Brook JR van Donkelaar A Martin RV Ambient PM2.5, O₃, and NO₂ Exposures and Associations with Mortality over 16 Years of Follow-Up in the Canadian Census Health and Environment Cohort (CanCHEC). Environ Health Perspect (2015) 123:1180–6. 10.1289/ehp.1409276 26528712 PMC4629747

[68] Le Y Ren J Shen J Li T Zhang CF. The Changing Gender Differences in Life Expectancy in Chinese Cities 2005-2010. PLOS ONE (2015) 10:e0123320–11. 10.1371/journal.pone.0123320 25875494 PMC4395256

[69] Rochelle TL Yeung DKY Bond MH Li LMW. Predictors of the Gender gap in Life Expectancy across 54 Nations. Psychol Health Med (2015) 20:129–38. 10.1080/13548506.2014.936884 25005485

[70] Aburto JM Beltrán-Sánchez H García-Guerrero VM Canudas-Romo V. Homicides in Mexico Reversed Life Expectancy Gains for Men and Slowed Them for Women, 2000–10. Health Aff (2016) 35:88–95. 10.1377/hlthaff.2015.0068 PMC5453309 26733705

