# Supplementary material for: Associations Between Gender Gaps in Life Expectancy, Air Pollution, and Urbanization: A Global Assessment With Bayesian Spatiotemporal Modeling
Source: Int J Public Health. 2023 May 10;68:1605345. doi: 10.3389/ijph.2023.1605345 (PMC10207345; doi:10.3389/ijph.2023.1605345)
Supplement: Supplementary file 1 [file DataSheet1.docx]

## Detailed methods

### *Spatiotemporal trends in GGLE*

To model GGLE, a series of Bayesian spatiotemporal models are proposed (Table S1), where for each country (*i* = 1, 2, ..., 134) and year step (*t* = 1960,1961, ..., 2018), the GGLE, *y*_it_, was modeled by $y_{it}\sim Normal (\mu_{it},\sigma_{e}^{2})$. Here, $\mu_{it}$ denotes the expected value of $y_{it}$ and $\sigma_{e}^{2}$ measures the variance of $y_{it}$. The $\mu_{it}$ could be calculated as the following formula (model M0s):

$\mu_{it}= \alpha+s_{i}+ \theta_{t}+ \delta_{it}$ (2)

In Eq.(2), $\alpha$ is an intercept that measures the overall $y_{\mathrm{it}}$ during the study period (1960 – 2018); The spatial term $s_{i}$, denotes the spatial random effect capturing the spatial dependency of $y_{it}$. We note that $s_{i}$ captures the overall spatial random effects common from 1960 to 2018, describes the difference between $y_{it}$ in the *i*-th country or region relative to the global average level, $\alpha$. When $s_{i}$>0, it indicates that the $y_{it}$ in country i is higher than overall $y_{it}$ across the whole study period. The term $\theta_{t}$ denotes a dynamic temporal trend that captures the overall temporal trend common to all countries. The $\delta_{it}$ is a space-time interaction random effect, representing a vector that varies through space and time (Johnson et al. 2021). The term $\delta_{it}$ allows spatial pattern changes from one time frame to another and temporal trend varies from one country to another.

Prior distributions are to be assigned for all unknown model parameters in model M0s. The term $s_{i}$ is defined widely as a Besag-York-Mollié (BYM) model to incorporate spatial autocorrelation in $s_{i}$ (Besag et al. 1991). Spatial autocorrelation is present when observations in adjacent countries have similar data values. To account for spatial autocorrelation can avoid inaccurate parameter estimates (Lym 2021). In the classical BYM model, the spatial effect $s_{i}$ is decomposed into the spatially structured component $u_{i}$ and unstructured component $\upsilon_{i}$, it is not clear to see how these two components are distinguished independently from each other (Yu et al. 2022). This is a well-known non-identifiability problem. We used a modified Besag-York-Mollié (BYM) model (Riebler et al. 2016) known as the BYM2 model (formula 3) to address the identifiability and scaling to generate clearly interpretable parameters.

$s_{i}=\upsilon_{i}+u_{i}= \frac{1}{\sqrt{\tau_{s}}}(\sqrt{\varphi}u_{i}^{*}+\sqrt{1-\varphi}\upsilon_{i}^{*})$ (3)

In Eq.(3), $\tau_{s}$ represents the overall precision for the spatial effect, $s_{i}$; $u_{i}^{*}$ and $\upsilon_{i}^{*}$ are the scaled structured spatial random effect and scaled unstructured spatial random effect, respectively; The term $\varphi\in[0,1]$ is the mixing parameter measuring how much of the marginal variance is explained by the structured effect. The magnitude of $\varphi$represents the spatial autocorrelation degree. When the value of $\varphi$ equal to 0, it indicates no spatial autocorrelation exists, while $\varphi$ close to 1 indicates strong spatial autocorrelation. When $\varphi=0$, the model reduces to pure over-dispersion, whilst when $\varphi=$1 the model reduces to the Besag model, i.e., only spatially structured effect. The BYM2 allows the hyperparameters $\tau_{s}$ and $\varphi$ to be mathematically interpretable and not confounded in the BYM (Riebler et al. 2016; Simpson et al. 2017).

We followed the procedures available elsewhere (Simpson et al. 2017), and Penalized Complexity (PC) prior was used for hyperparameters $\tau_{s}$ and $\varphi$ to overcome the shortcomings of non-informative reference priors used in the BYM model (Lym 2021). In particular, we used $Prob(\frac{1}{\sqrt{\tau_{s}}}>\frac{0.3}{0.31})=0.01$ and $Prob(\varphi<0.5)=0.7$ to reflect the assumption that $\tau_{s}$ is less than 2 with a probability of 0.99 and $u_{i}^{*}$ explains least of the variance (Baquero et al. 2018). Specifically, we used $U=0.5$ and $\alpha=2/3$ for the prior of $\varphi$ (Riebler et al. 2016). N(0,1) is assigned to $logit(\varphi)=log(\frac{\varphi}{1-\varphi})$ (Morris et al. 2019). Further, to investigate the sensitivity of our results due to different priors assigned to hyperparameters $\tau_{s}$ and $\varphi$, we also used $Prob(\frac{1}{\sqrt{\tau_{s}}}>\frac{0.1}{0.31})=0.01$ and $Prob(\varphi<0.1)=0.$9, implying that the fitted model with strong penalizing priors. Vague priors $\tau_{s}=logGamma(1,0.001)$ and $Prob(\varphi<0.5)=0.$5 were also used for sensitivity analysis.

In this study, the temporally structured effect $\theta_{t}$ is modeled as a first-order conditional autoregressive random walk (RW1) to incorporate temporal autocorrelation. The space-time interaction random effect $\delta_{it}$ utilized all four types of space-time interactions followed by Knorr-Held (2000), and we used Type 4 in the final model because it best fit our data. The Knorr-Held Type 4 space-time interaction random effect allows the random walk $\theta$ to interact with the intrinsic autoregression $s$ (Knorr-Held 2000). This prior for $\delta_{it}$ assumed temporal trend in country *i* is similar to the average trend in adjacent countries (Knorr-Held 2000). A noninformative prior N(0,1000) is assigned to $\alpha$.

We used *poly2nb* function in *spdep* R package to generate a neighborhood graph based on countries with contiguous boundaries. In order to obtain a fully connected neighborhood graph to meet the need of BYM2 prior, we manually added contiguity between ‘islands’ (e.g., U.K., Australia, Japan) and the rest of the countries as a prior study used (Morris et al. 2019).

### *Bayesian spatiotemporal ecological regression*

A Bayesian spatiotemporal ecological regression was developed to investigate the impacts of air pollution and urbanization on GGLE and gender-specific life expectancy while adjusting for confounding factors and spatiotemporal variation. As an extension of model M0s, the variables of PM and UP (two interested variables) were considered, adjusting for confounding factors like poverty (P), education (E), calorie supply (C), and smoking (S). The new model was defined as the following formula (model M1s):

$y_{it}=\alpha+s_{i} +\theta_{t}+ \delta_{it}+\beta_{1}{ln\mathrm{PM}}_{\mathrm{it}}+\beta_{2}{ln\mathrm{UP}}_{\mathrm{it}} + \beta_{3}{lnP}_{\mathrm{it}}+ \beta_{4}{lnE}_{\mathrm{it}} +\beta_{5}{lnC}_{\mathrm{it}}+ \beta_{6}{lnS}_{\mathrm{it}}$ (4)

In Eq.(4), $\beta_{1}$, $\beta_{2}$, ..., $\beta_{6}$ refer to the corresponding regression coefficients of pwPM_2.5_, urbanization, poverty, education, supply of calories, and smoking, respectively, measuring the effects of these six variables across 134 overall countries on y (GGLE, LEm, and LEf). A previous study found that the effects of air pollution and urbanization on LE and gender-specific LE may be spatially varied (Wang et al. 2021). Model M1s is modified to allow each region to present its own regression coefficient to examine the spatially varied impacts of pwPM_2.5_ and urbanization on GGLE and gender-specific LE. In this paper, a total of 134 countries are divided into six regions based on the definition of the World Health Organization (WHO) (i.e., Africa, Americas, Eastern Mediterranean, Europe, South-East Asia, and Western Pacific), to investigate the region-specific effects of pwPM_2.5_ and urbanization on GGLE and gender-specific life expectancy, the modified regression model is shown as follow (model M2s):

$y_{it}=\alpha+s_{i} \theta_{t}+\delta_{it}+\sum_{j=1}^{n} \beta_{1j}{ln\mathrm{PM}}_{\mathrm{it}}+\sum_{j=1}^{n} \beta_{2j}{lnUP}_{\mathrm{it}}+\beta_{3}{lnP}_{\mathrm{it}}+\beta_{4}{lnE}_{\mathrm{it}}+\beta_{5}{lnC}_{\mathrm{it}}+\beta_{6}{lnS}_{\mathrm{it}}$ (5)

In Eq. (5), $\beta_{1j}$ and $\beta_{2j}$ represent the regression coefficients of influencing factors in a different region, j = 1, 2, …, n (n = 6).

Prior to building a multivariable Bayesian spatiotemporal ecological regression model, we tested multi-collinearity among all the confounding factors using variance inflation factors (VIFs) (Fox 2015). Finally, the variables with a VIF value below 3 (Ribeiro et al. 2018) were selected for the final model to examine the effects of air pollution and urbanization on GGLE and gender-specific life expectancy. The deviance information criterion (DIC) and Watanabe-Akaike information criterion (WAIC) were used as diagnostic tools to compare the fitness among different models. Lower DIC and WAIC scores suggest a better model fit. The analyses were performed in the R software with the integrated nested Laplace approximation (INLA) packages (Rue et al. 2009; Blangiardo et al. 2013), and car packages (Fox and Weisberg 2019).

A sensitivity analysis is conducted to examine the robustness of our findings regarding the priors used for hyperparameters $\tau_{s}$ and $\varphi$. The three pairs of priors for these two hyperparameters were in our analysis for all models (Table S2). We ran the sensitivity analysis proposed in this paper based on different recommendations from the literature (Baquero et al. 2018). Similar estimates were found in Table S3. In sum, the sensitivity analysis indicated that the posterior estimates and resulting DIC scores and WAIC scores were both robust regarding the different prior distributions.

**References**

Baquero OS, Ferreira F, Robis M, et al (2018) Bayesian spatial models of the association between interpersonal violence, animal abuse and social vulnerability in São Paulo, Brazil. Preventive Veterinary Medicine 152:48–55. https://doi.org/10.1016/j.prevetmed.2018.01.008

Besag J, York J, Mollié A (1991) Bayesian image restoration, with two applications in spatial statistics. Annals of the institute of statistical mathematics 43:1–20

Blangiardo M, Cameletti M, Baio G, Rue H (2013) Spatial and spatio-temporal models with R-INLA. Spatial and Spatio-temporal Epidemiology 4:33–49. https://doi.org/10.1016/j.sste.2012.12.001

Fox J (2015) Applied Regression Analysis and Generalized Linear Models. Sage Publications, Thousand Oaks, California.

Fox J, Weisberg S (2019) An R Companion to Applied Regression, Third edition. Sage, Thousand Oaks, California.

Johnson DP, Ravi N, Braneon CV (2021) Spatiotemporal Associations Between Social Vulnerability, Environmental Measurements, and COVID-19 in the Conterminous United States. GeoHealth 5:e2021GH000423. https://doi.org/10.1029/2021GH000423

Knorr-Held L (2000) Bayesian modelling of inseparable space-time variation in disease risk. Statistics in Medicine 19:2555–2567. https://doi.org/10.1002/1097-0258(20000915/30)19:17/18<2555::AID-SIM587>3.0.CO;2-#

Lym Y (2021) Exploring dynamic process of regional shrinkage in Ohio: A Bayesian perspective on population shifts at small-area levels. Cities 115:103228. https://doi.org/10.1016/j.cities.2021.103228

Morris M, Wheeler-Martin K, Simpson D, et al (2019) Bayesian hierarchical spatial models: Implementing the Besag York Mollié model in stan. Spatial and Spatio-temporal Epidemiology 31:100301. https://doi.org/10.1016/j.sste.2019.100301

Ribeiro AG, Baquero OS, Freitas CU de, et al (2018) Incidence and mortality risk for respiratory tract cancer in the city of São Paulo, Brazil: Bayesian analysis of the association with traffic density. Cancer Epidemiology 56:53–59. https://doi.org/10.1016/j.canep.2018.07.005

Riebler A, Sørbye SH, Simpson D, Rue H (2016) An intuitive Bayesian spatial model for disease mapping that accounts for scaling. Stat Methods Med Res 25:1145–1165. https://doi.org/10.1177/0962280216660421

Rue H, Martino S, Chopin N (2009) Approximate Bayesian inference for latent Gaussian models by using integrated nested Laplace approximations. Journal of the royal statistical society: Series b (statistical methodology) 71:319–392

Simpson D, Rue H, Riebler A, et al (2017) Penalising Model Component Complexity: A Principled, Practical Approach to Constructing Priors. Statistical Science 32:1–28. https://doi.org/10.1214/16-STS576

Wang S, Ren Z, Liu X, Yin Q (2021) Spatiotemporal trends of life expectancy, economic growth, and air pollution: A 134 countries investigation based on Bayesian modeling. Social Science & Medicine 114660. https://doi.org/10.1016/j.socscimed.2021.114660

Yu H, Jiang S, Huang H (2022) Spatio-temporal parse network-based trajectory modeling on the dynamics of criminal justice system. Journal of Applied Statistics 49:1979–2000. https://doi.org/10.1080/02664763.2021.1887101

## Table S1. Description of fitted models.

| Model | Formula |
| --- | --- |
| M0n | ${lnLE}_{it}=\alpha+\theta_{t}+ \delta_{it}$ |
| M0s | ${lnLE}_{it}=\alpha+s_{i} +\theta_{t}+ \delta_{it}$ |
| M1n | ${lnLE}_{it}=\alpha+\theta_{t}+ \delta_{it}+\beta_{1}{ln\mathrm{PM}}_{\mathrm{it}}+ \beta_{2}{ln\mathrm{UP}}_{\mathrm{it}} + \beta_{3}{lnP}_{\mathrm{it}}+ \beta_{4}{lnE}_{\mathrm{it}} +\beta_{5}{lnC}_{\mathrm{it}}+ \beta_{6}{lnS}_{\mathrm{it}}$ |
| M1s | ${lnLE}_{it}=\alpha+s_{i} +\theta_{t}+ \delta_{it}+\beta_{1}{ln\mathrm{PM}}_{\mathrm{it}}+ \beta_{2}{ln\mathrm{UP}}_{\mathrm{it}} + \beta_{3}{lnP}_{\mathrm{it}}+ \beta_{4}{lnE}_{\mathrm{it}} +\beta_{5}{lnC}_{\mathrm{it}}+ \beta_{6}{lnS}_{\mathrm{it}}$ |
| M2s | ${lnLE}_{it}=\alpha+s_{i} +\theta_{t}+ \delta_{it}+\sum_{j=1}^{n} \beta_{1j}{ln\mathrm{PM}}_{\mathrm{it}}+\sum_{j=1}^{n} \beta_{2j}{lnUP}_{\mathrm{it}} +\beta_{2}{ln\mathrm{UP}}_{\mathrm{it}} + \beta_{3}{lnP}_{\mathrm{it}}+ \beta_{4}{lnE}_{\mathrm{it}} +\beta_{5}{lnC}_{\mathrm{it}}+ \beta_{6}{lnS}_{\mathrm{it}}$ |

## Table S2. Prior distribution for hyperparameters $\tau_{s}$ and $\varphi$.

|  | $\tau_{s}$ | $\varphi$ |
| --- | --- | --- |
| Prior1 | $Prob(\frac{1}{\sqrt{\tau_{s}}}>\frac{0.3}{0.31})=0.01$ | $Prob(\varphi<0.5)=0.7$ |
| Prior2 | $Prob(\frac{1}{\sqrt{\tau_{s}}}>\frac{0.1}{0.31})=0.01$ | $Prob(\varphi<0.1)=0.$9 |
| Prior3 | $\tau_{s}=logGamma(1,0.001)$ | $Prob(\varphi<0.5)=0.$5 |

## Table S3. Sensitivity analysis for different prior distribution of hyperparameters $\tau_{s}$ and $\varphi$ that modeling GGLE (posterior means of parameters with 95% credible interval)

| Fixed effects | Prior1 | Prior2 | Prior3 |
| --- | --- | --- | --- |
| (Intercept) | 7.861  (4.312,11.406) | 7.972  (4.433, 11.508) | 7.799  (4.240, 11.356) |
| pwPM2.5 | 0.085*  (0.049, 0.120) | 0.083*  (0.047, 0.119) | 0.085*  (0.049, 0.121) |
| urbanpop | 0.020  (-0.310, 0.349) | 0.085  (-0.245, 0.414) | -0.012  (-0.340, 0.314) |
| poverty | -0.046*  (-0.068, -0.025) | -0.046*  (-0.068, -0.025) | -0.047*  (-0.068, -0.025) |
| education | -3.161*  (-3.768, -2.552) | -3.206*  (-3.815, -2.596) | -3.137*  (-3.740, -2.532) |
| calories supply | 0.621*  (0.260, 0.982) | 0.614*  (0.253, 0.975) | 0.626*  (0.266, 0.986) |
| smoking | 0.872*  (0.648, 1.100) | 0.842*  (0.619, 1.069) | 0.884*  (0.664, 1.109) |
| Random effects (hyperparameters) |  |  |  |
| $\tau_{e}$ (Precision of Gaussian error) | 83.733  (75.602,92.429) | 83.600  (75.590,92.111) | 83.769  (75.724,92.407) |
| $\tau_{s}$ (marginal precision of BYM2) | 0.196  (0.099,0.344) | 0.373  (0.229,0.576) | 0.104  (0.043,0.199) |
| $\varphi$ (mixing parameter of BYM2) | 0.077  (0.004,0.353) | 0.031  (0.009,0.073) | 0.041  (0.007,0.105) |
| $\tau_{\theta}$ (precision of $\theta_{t}$) | 1199.562  (528.622,2283.324) | 1256.506  (494.398,2519.318) | 1262.874  (502.146,2391.998) |
| $\tau_{\delta}$ (precision of space-time interaction) | 7.691  (7.000,8.425) | 7.694  (7.000,8.434) | 7.693  (7.000,8.428) |
| Goodness of fit |  |  |  |
| DIC | -2435.95 | -2428.72 | -2435.14 |
| WAIC | -2580.37 | -2576.40 | -2587.32 |
